# Mechanical Reinforcement by Microalgal Biofiller in Novel Thermoplastic Biocompounds from Plasticized Gluten

**DOI:** 10.3390/ma12091476

**Published:** 2019-05-07

**Authors:** Riccardo Ciapponi, Stefano Turri, Marinella Levi

**Affiliations:** 1 INSTM–National Interuniversity Consortium of Materials Science and Technology, Via G. Giusti 9, 50121 Firenze, Italy; stefano.turri@polimi.it; 2 Department of Chemistry, Materials and Chemical Engineering “Giulio Natta”, Politecnico di Milano, Piazza Leonardo da Vinci 32, 20133 Milano, Italy; marinella.levi@polimi.it

**Keywords:** microalgae, bioplastics, gluten, biofiller, plasticizers, SaltGae

## Abstract

The aim of this work was to develop new bioplastic compounds from wheat gluten, biobased plasticizers (glycerol, octanoic acid and 1,4-butanediol), and microalgal biomass as a filler. The effects of the composition on tensile properties, thermal stability, and water sensitivity were investigated. Microalgal biomass was added with the selected quantities: 10, 20, and 30 per hundred parts (php). Mechanical mixing of the components, i.e., gluten, plasticizer, and microalgae, was followed by molding in a hot press. Microlgal filler improved mechanical properties of the plasticized gluten material: in samples plasticized with 1,4-butanediol, 30 php of biomass increased the tensile modulus by nearly one order of magnitude, from 36.5 MPa to 273.1 MPa, and it also increased the tensile strength from 3.3 MPa to 4.9 MPa. The introduction of microalgal biomass slightly increased the surface sensitivity against water: 30 php of biomass reduced the water contact angle from 41° to 22° in samples plasticized with glycerol, but the biomass lowered the overall water absorption kinetics for material with each plasticizer. Microalgal biomass proved therefore to be an interesting sustainable resource with which to develop materials based on gluten, in particular to increase the mechanical properties of the compounds without reducing thermal stability or water resistance.

## 1. Introduction

The treatment of wastewater with microalgae is a promising technology for removing organic pollutants from industrial, agricultural, and urban effluents in an efficient and affordable way [1,2]. In particular, the ability of microalgae to remove N and P, heavy metals, and to reduce biochemical oxygen demand (BOD) from water is an important advantage of this method [3].

The valorization of the biomass after the main purification process is an important aspect to be considered, in order to generate a resource instead of waste from a circular economy perspective [4]. In previous studies, microalgae proved to be a good intermediate to obtain bioethanol [5], carotenoids, and fatty acids [6].

Furthermore, the possibility of using microalgae as fillers has been investigated in fossil-based polymers [7], in blends of fossil-based and bioplastics [8], in renewable and biodegradable plastics [9], and in gel systems based on proteins [10].

Wheat gluten is an important source of proteins. Being a by-product of the starch industry, it is widely available, cheap, and fully biodegradable. Among other possible uses, gluten has been extensively tested to produce renewable thermoplastic materials [11,12]. In particular, through combination of extrusion and compression molding, it is possible to obtain thermoplastic gluten films [13]. One limitation of gluten is its thermal induced crosslinking by S–S bonds, increasing the material brittleness and limiting its possible final applications, a phenomenon which may be minimized by reducing the processing temperature and time [14]. In order to do that, plasticizers must be used, but they lower the mechanical properties [15]. Materials obtained from gluten are considered a promising source with which to produce sustainable packaging [16], possibly also including antimicrobial agents in the matrix [17].

Gluten has been used as a matrix to form several composites. Works have been published concerning the production of composites with non-renewable materials, such as nylon [18], montmorillonite [19], and kaolin [20].

The effect of renewable fillers on mechanical properties of plasticized gluten has been studied; in particular, lignin nanoparticles [21], fish scales [22], olive pomace [23], and banana fiber [24] have been proposed.

In this work, microalgal biomass (from *Spirulina platensis*) was investigated as a renewable reinforcing biofiller for the realization of innovative protein-based thermoplastic compounds from plasticized wheat gluten.

## 2. Materials and Methods

Wheat gluten, glycerol (GLY), octanoic acid (OA), and 1,4-butanediol (BU) were supplied by Sigma Aldrich. Microalgal biomass, composed by freeze dried *Spirulina platensis* (SP), was kindly supplied by Archimede Ricerche Srl. Before compounding, the biomass was ground in a ball mill using zirconia spheres for 24 h, in order to reduce the size of the aggregates.

Gluten compounds were prepared by mechanical mixing with a Brabender internal mixer, preheating the mixture to 80 °C and always keeping the temperature below 100 °C. The minimum time for mixing was 2 min, while torque and temperature were monitored: a plateau of the applied torque was considered an indication of complete mixing. Microalgal biomass was added in amounts of 10, 20, and 30 per hundred parts (php) with respect to the total amount of gluten and plasticizer.

The bioplastic material obtained was then shaped in slabs of 1 mm thickness by compression molding in a hot press (T = 120 °C) applying a pressure of 40 bar for 10 min in an aluminum mold.

Differential scanning calorimetry (DSC) was performed with a DSC 823e (Mettler-Toledo, Columbus, OH, USA) by performing three runs: from −20 °C to 150 °C at 20 °C/min, from 150 °C to −100 °C at −20 °C/min, and from −100 °C to 150 °C at 20 °C/min, in order to determine the thermal transitions.

Thermogravimetric analysis (TGA) was performed with a Q500 TGA system (TA Instruments, New Castle, DE, USA), from ambient temperature to 800 °C at a scan rate of 10 °C/min, both in air and nitrogen.

Mechanical tests were performed with a Zwick/Roell Z010 (Zwick Roell, Ulm, Germany) at room temperature, according to ASTMD638-10 [25].

Optical contact angle (OCA) tests were performed with an OCA 20 instrument (Data physics Co., San Jose, CA, USA), equipped with a CCD photo camera and with a 500 μL Hamilton syringe, using water as a testing liquid.

Water vapor transmission rate (WVTR) was measured by the weighting cups method (ASTM ES96/ES96M-16 [26]). Slabs were cut into circular shapes with a diameter of 41 mm and used as a membrane through which water, contained in a cup, can evaporate. Cups were kept in a thermostatic room at T = 20 °C and RH = 40%. WVTR is defined as the mass loss versus time, normalized by the cross section of the sample.

Scanning electron microscopy (SEM) was performed with a Carl Zeiss EVO 50 extended pressure scanning electron microscope (Carl Zeiss, Oberkochen, Germany).

## 3. Results

### 3.1. Plasticization of Gluten

**Figure 1:**
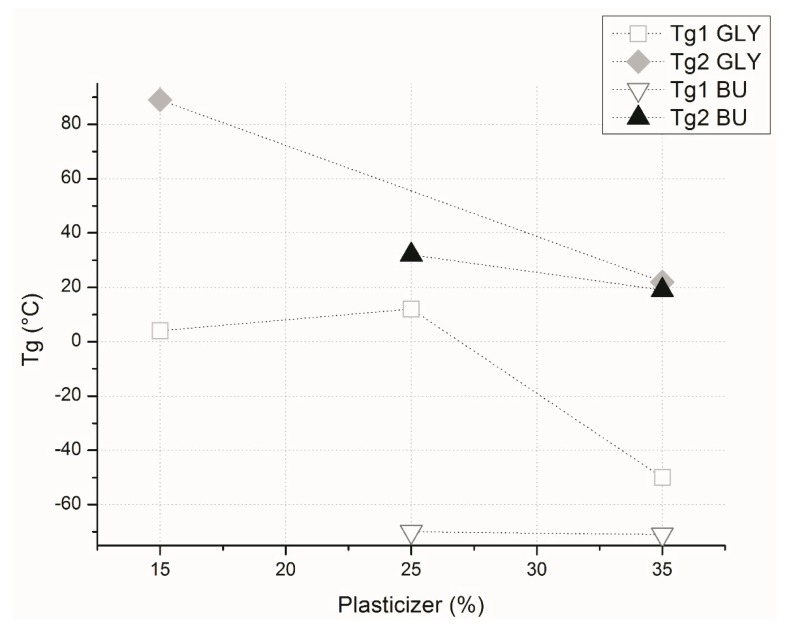
Glass transition temperatures of the materials vs. plasticizer content. Two glass transition temperatures were present in the materials: the lower one is plotted as T_g_1, while the higher one is plotted as T_g_2 for both glycerol and 1,4-butanediol.

Wheat gluten was plasticized with different amounts of glycerol and 1,4-butanediol, a compound that can be obtained by biosynthesis [27], in order to reduce its glass transition temperature (T_g_) and improve processability. A comparison was also made with octanoic acid: although the solubility of such fatty acids is limited, it proved to be able to increase water vapor barrier properties [28]. Each plasticizer was separately added, forming three mixtures with different amounts of plasticizer: 15%, 25%, and 35% (*w*/*w*). Figure 1 shows the effect of type and amount of plasticizers on the glass transition temperatures of the compound materials.

Figure 1 shows that the addition of glycerol and butanediol results in both cases in the formation of a phase rich in plasticizer with a low glass transition temperature (T_g_1), and one rich in gluten with a high glass transition temperature (T_g_2) [11,29]; the glass transition temperature of wheat gluten without plasticizer is 112 °C. By increasing the amount of plasticizer, the glass transition temperature of the gluten-rich phase was lowered, as expected. Interestingly, the presence of butanediol did not affect the T_g_ of the plasticizer-rich phase, while glycerol did so after 25% content.

### 3.2. Tensile Tests

**Figure 2:**
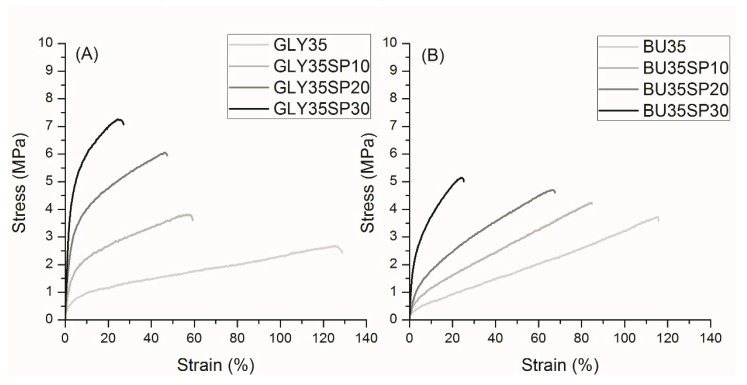
Stress–strain curves of gluten samples plasticized with glycerol (**A**) and 1,4-butanediol (**B**) with 0, 10, 20, and 30 php of microalgal (*Spirulina platensis*) biomass.

After molding in a hot press (T = 120 °C), the samples were cut into dumbbell shapes and tested. Results of the tensile tests on microalgae filled gluten specimens are shown in Figure 2.

Figure 2 shows quite clearly that the addition of microalgal biomass significantly increased the elastic modulus and the tensile strength of the plasticized gluten compounds, while progressively lowering their elongation at break. On the other hand, the introduction of the plasticizer, which was necessary in order to allow a thermoplastic processing of the compound, led to a very soft, unfilled material with quite poor mechanical properties. Table 1 shows the numerical results of the tensile tests.

Increasing the amount of biofiller led to an increase in the tensile modulus (E_t_) and the tensile strength (σ_B_), while the elongation at break (ε_B_) was lowered, with a significant difference for samples with 30 php of microalgal biomass. Toughness, estimated by the area under the curve, had no clear trend, however, in some cases, especially with 1,4-butanediol plasticizer, it increased with respect to the unfilled material (like BU35SP10 and BU35SP20).

### 3.3. Thermogravimetric Analysis

**Figure 3:**
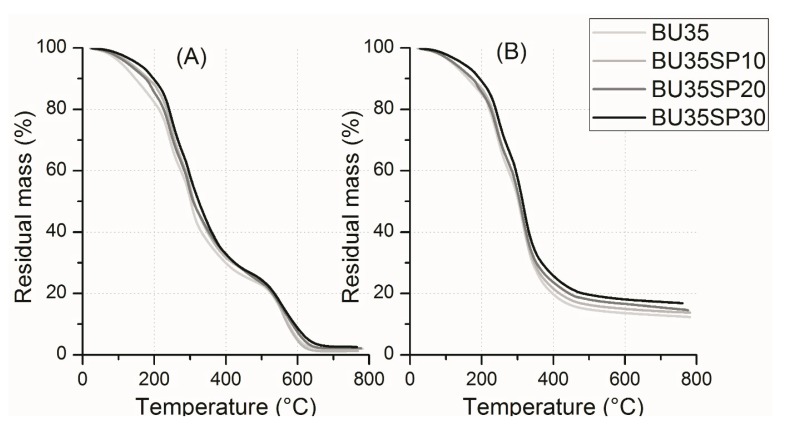
Thermogravimetric analysis (TGA) curves between T_amb_ and 800 °C of samples plasticized with 1,4-butanediol with 0, 10, 20, and 30 php of microalgal biomass, in air (**A**) and nitrogen (**B**).

Results of thermogravimetric analysis (TGA) analyses are shown in Figure 3.

Figure 3 shows that the increasing biomass content slightly increased the residual weight of the TGA curves, with the main difference in the devolatilization stage (cleavage of S–S, O–N, and O–O in the protein molecules) that started around 200 °C, in agreement with previously reported results [19,23,30]. More interestingly, the thermal stability of the compound in the lower temperature range (below +150 °C) was improved by the presence of the microalgal biofiller. In air, the residual mass was completely volatilized after 650 °C, a phenomenon not observed in nitrogen and therefore probably related to the oxidation of char residues, as previously observed [31].

### 3.4. Contact Angle, Transmisison Rate, and Kinetic Absorption with Water

In order to observe the sensitivity of gluten materials towards water, several tests were performed. The results for optical contact angle and, in some cases, the water vapor permeability are summarized in Table 2.

Table 2 shows that by increasing the amount of microalgal biomass, the water contact angle decreased too, probably due to the hydrophilic nature of the biomass. The addition of 20 php of microalgal biomass did not affect WVTR, while the diffusion coefficient, tested only for samples plasticized with 1,4-butanediol, was slightly lowered.

**Figure 4:**
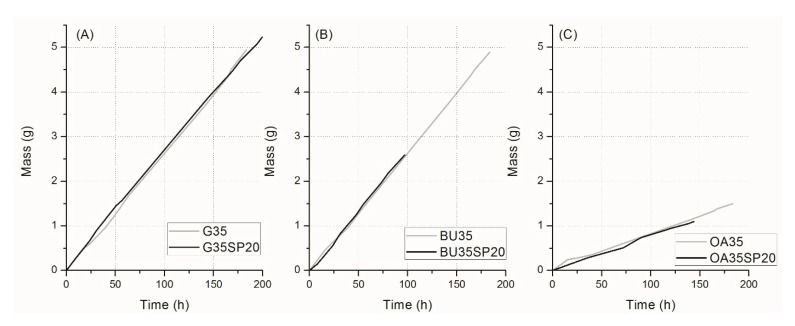
Mass loss of water through films of gluten plasticized with 35% of glycerol (**A**), 1,4-butanediol (**B**), and octanoic acid (**C**), with and without 20 php of microalgal biomass.

The water barrier properties were tested with the weighting cup method, and results are shown in Figure 4.

Figure 4 shows that the presence of the biomass did not significantly change the barrier properties of the films, which was instead significantly affected by the chemical nature of the plasticizer, as previously reported [14,28,32]. Indeed, Figure 4 also shows the behavior of films plasticized with octanoic acid, for comparison. The latter plasticizer, being characterized by a long paraffinic chain, is much more efficient than the others to decrease the permeability of the film against water.

**Figure 5:**
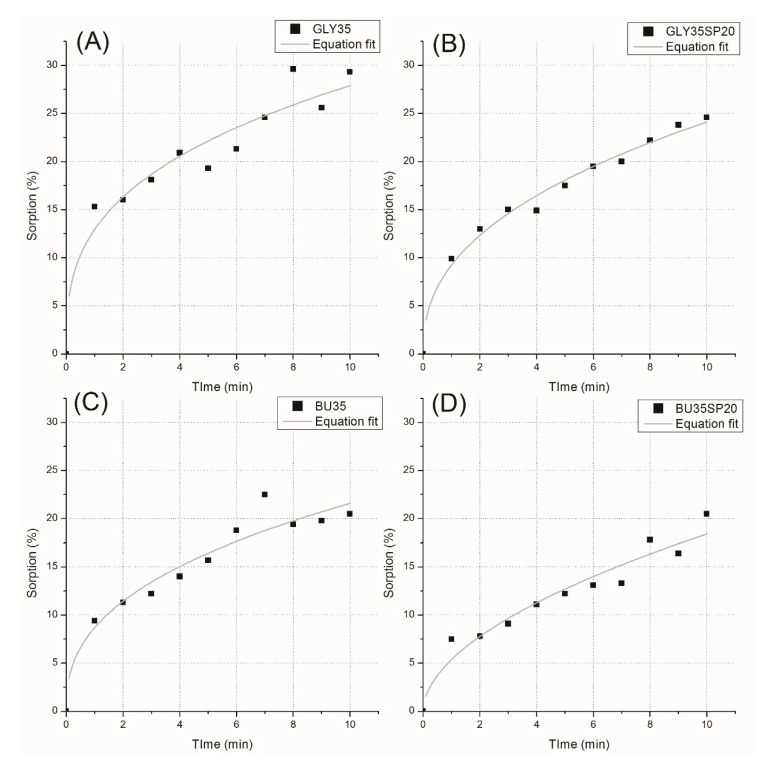
Kinetic water absorption test results with a sample of gluten plasticized with 35% of glycerol (**A**) and with the addition of 20 php of microalgal biomass (**B**); with 35% of 1,4-butanediol (**C**) and with the addition of 20 php of microalgal biomass (**D**).

Figure 5 shows the results of the kinetic water absorption tests.

According to Equation (1),

(1) $$y=A\;\cdot x^{n}$$

the *A* and *n* parameters were calculated for the two materials. Results of those calculations, with their respective R^2^, are reported in Table 3.

Table 3 shows that samples plasticized by butanediol presented an exponent of the sorption curve very close to 0.5, which is an indication of Fickian diffusion [33]. Figure 5 and Table 3 show instead that samples plasticized with glycerol showed a faster absorption rate. The addition of 20 php of microalgal biomass slowed down the absorption kinetics of both materials.

### 3.5. Scanning Electron Microscopy

**Figure 6:**
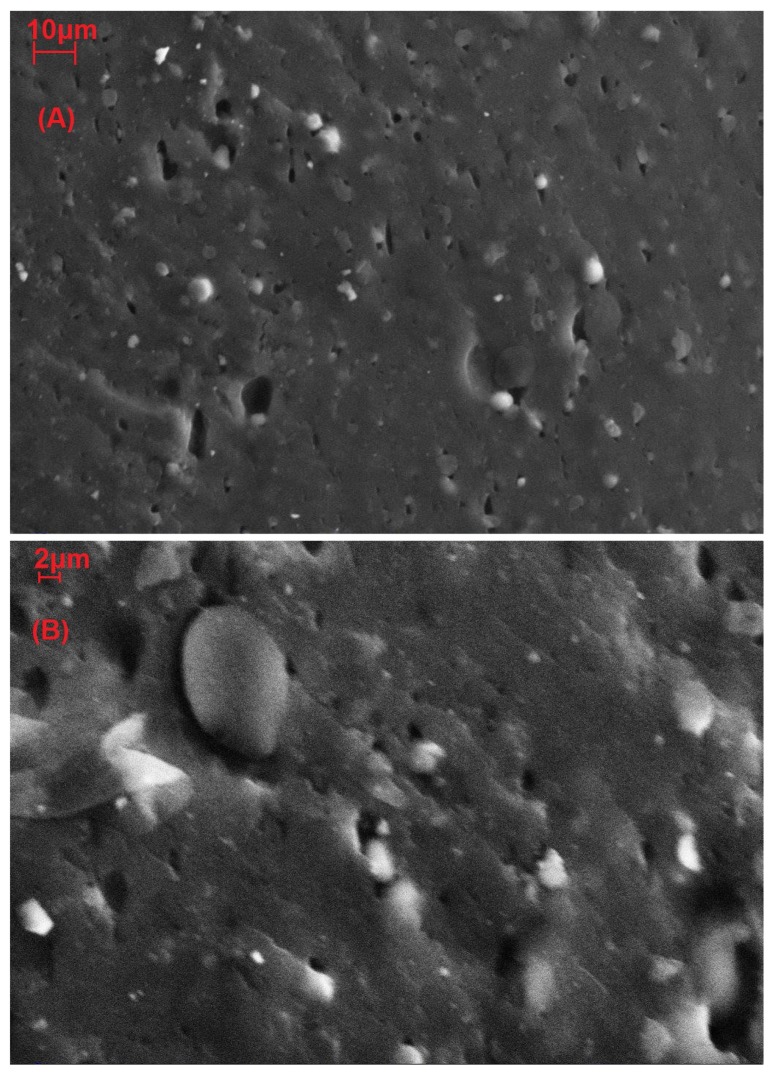
Scanning electron microscope image of wheat gluten plasticized with 35% of 1,4-butanediol and 10 php of microalgal biomass, magnifications 2000× (**A**) and 5000× (**B**).

**Figure 7:**
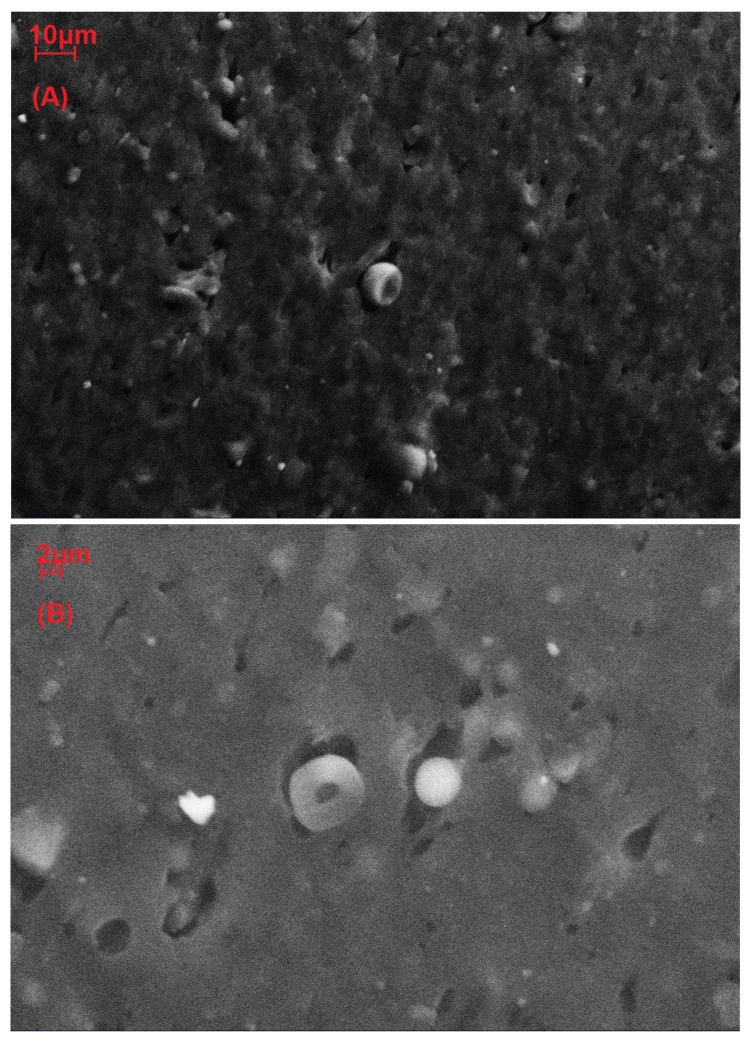
Scanning electron microscope image of wheat gluten plasticized with 35% of 1,4-butanediol and 20 php of microalgal biomass, magnifications 2000× (**A**) and 5000× (**B**).

Figure 6 and Figure 7 show the morphology of samples plasticized with 35% 1,4-butanediol with 10 and 20 php of microalgal biomass (fractured surfaces).

Figure 6 shows that the biomass particles were rounded with a size distribution of bigger particles, about 2–3 μm of diameter, and some smaller that were not visible. Some voids were present, showing that the adhesion of the larger particles to the gluten matrix seemed rather limited.

Figure 7 shows a fracture surface that is less regular than what was observed in Figure 6. A bigger, cleaved particle is visible, probably implying a lower resistance of big (6–8 μm) aggregates towards fracture. The morphology of the sample with 20 php of biomass (Figure 7) was still similar to the one of sample with 10 php of biomass (Figure 6).

## 4. Discussion

Mechanical test results (Table 1) clearly show that microalgal biomass may act as a reinforcing filler on plasticized gluten thermoplastics. Proteins in the microalgae and gluten are probably able to interact, promoting good adhesion between the biofiller and the plasticized matrix, provided that the biofiller particles are small enough. This good interaction between gluten and biomass was also observed in SEM images of cold fracture surfaces (Figure 6 and Figure 7). The dispersion method, i.e., mechanical mixing, was also effective, showing only few aggregates bigger than 6 μm that could limit or lower the improvement of mechanical properties. The reinforcing effect on tensile strength was comparable to the effect of olive pomace, while the effect on tensile modulus was more conspicuous for the microalgae [23].

While the microalgal biomass was more hydrophilic than the matrix, as shown in Table 2, the presence of the biofiller particles slowed down the water absorption kinetics of the material in the selected timeframe. Indeed, it was previously reported that the presence of reinforcing fillers in gluten changes the absorption behavior of the material [34].

Protein films tend to have a high water permeability, compared to fossil-based polymeric films [35]. The considered biomass (*Spirulina platensis*) has a high content of proteins [36], therefore WVTR of gluten was not affected by the presence of this type of biomass.

## 5. Conclusions

Both glycerol and 1,4-butanediol can be used as effective plasticizers for wheat gluten, allowing for an easy thermoplastic processing, but at the same time significantly decreasing both its T_g_ and mechanical properties. Both substances can be obtained from renewable sources, allowing the production of a sustainable material with 100% renewable carbon.

It was demonstrated that microalgal biomass can be successfully added as a reinforcing biofiller to plasticized wheat gluten thermoplastics. Microalgae effectively reinforced the protein-based material, increasing both the elastic modulus and the tensile strength, and synergistically even the toughness in some cases.

Microalgal biomass slightly improved the thermal stability of the compound in the processing temperature range (up to 120 °C).

The addition of the algal biofiller lowered the kinetic water absorption rate, which was also affected by the plasticizer, resulting in a lower rate with 1,4-butanediol with respect to glycerol.

Scanning electron microscopy showed a good dispersion of the biomass, with the presence of few aggregates with a diameter greater than 5 μm that were not able to reinforce the material, while the majority of the particles were smaller than 3 μm, effectively reinforcing the material, as confirmed by the stress–strain curves.

It is realistic to think that mechanical behavior could be further improved with a more efficient dispersive mixing of algae in the bioplastic matrix.

## Figures and Tables

**Table 1:** Elastic modulus (E_t_), elongation at break (ε_b_), stress at break (σ_b_), and toughness values of samples plasticized with 35% glycerol and 1,4-butanediol, with 0, 10, 20, and 30 php of microalgal biomass.

Sample	E_t_ (MPa)	ε_B_ (%)	σ_B_ (MPa)	Toughness (MJ∙m^−3^)
GLY35	44.1 ± 8.9	120.6 ± 13.7	2.6 ± 0.3	2.1 ± 0.4
GLY35SP10	112.6 ± 32.0	48.3 ± 16.4	3.5 ± 0.5	1.4 ± 0.5
GLY35SP20	217.6 ± 41.3	57.3 ± 13.0	5.1 ± 0.7	2.7 ± 0.7
GLY35SP30	307.0 ± 45.8	29.8 ± 5.4	6.5 ± 1.2	1.8 ± 0.4
BU35	36.5 ± 9.0	105.2 ± 13.8	3.3 ± 0.4	1.5 ± 0.6
BU35SP10	51.5 ± 11.3	82.1 ± 10.5	4.2 ± 0.6	2.2 ± 0.4
BU35SP20	94.0 ± 28.3	60.7 ± 14.6	4.7 ± 0.5	2.0 ± 0.4
BU35SP30	273.1 ± 59.0	22.2 ± 7.8	4.9 ± 0.9	1.0 ± 0.4

**Table 2:** Water contact angle, water vapor transmission rate, and water diffusion coefficient of samples plasticized with glycerol and 1,4-butanediol with 0, 10, 20, and 30 php of microalgal biomass.

Sample	CA (°)	WVTR (g∙h^−1^∙m^−2^)	Diffusion Coefficient (cm^2^∙s^−1^)
GLY35	41 ± 5	20.2	-
GLY35SP10	27 ± 3	-	-
GLY35SP20	24 ± 2	20.1	-
GLY35SP30	22 ± 3	-	-
BU35	32 ± 5	20.2	4.1 × 10^−7^
BU35SP10	34 ± 3	-	-
BU35SP20	35 ± 2	20.3	3.7 × 10^−7^
BU35SP30	29 ± 5	-	-

CA, contact angle; WVTR, Water vapor transmission rate.

**Table 3:** *A*, *n*, and R-square parameters for the water absorption equation for samples with 35% of glycerol and 1,4-butanediol, with and without 20 php of microalgal biomass.

Sample	*A*	*n*	R^2^
GLY35	13.1 ± 1.3	0.3 ± 0.1	0.83
GLY35SP210	9.2 ± 0.5	0.4 ± 0.1	0.97
BU35	8.7 ± 1.0	0.5 ± 0.1	0.87
BU35SP20	5.4 ± 0.8	0.5 ± 0.1	0.89
